# Fatty Acids from *Hermetia illucens* Larvae Fat Inhibit the Proliferation and Growth of Actual Phytopathogens

**DOI:** 10.3390/microorganisms8091423

**Published:** 2020-09-16

**Authors:** Elena Marusich, Heakal Mohamed, Yuriy Afanasev, Sergey Leonov

**Affiliations:** 1 School of Biological and Medical Physics, Moscow Institute of Physics and Technology, 141700 Dolgoprudny, Moscow Region, Russia; m.heakal@phystech.edu (H.M.); yurii.afanasev@phystech.edu (Y.A.); 2 Institute of Cell Biophysics of the Russian Academy of Sciences, 142290 Pushchino, Moscow Region, Russia

**Keywords:** black soldier fly, fatty acids, larvae extract, phytopathogenic bacteria, antibacterial activity

## Abstract

The rapid increase of plant diseases caused by bacterial phytopathogens calls for an urgent search for new antibacterials. Antimicrobial compounds of natural origin stand up as frontiers in the attempts of the antibiotic overuse replacement. With this in mind, the *Hermetia illucens (H. illucens)* larvae have recently gained attention as a promising approach to fulfill this need. This study aimed to isolate the active constituents of *H. illucens* larvae fat and to estimate its antimicrobial capacity. We discovered the best composition of extracting solution retaining the pronounced antimicrobial activity of the extract. Using gas chromatography-mass spectrometry (GC-MS), we identified the unique natural array of fatty acids as the major constituents of the acidified water-methanol extract (AWME) as having new antimicrobial potency. In standard turbidimetric assay, the minimum inhibitory concentration (MIC) of the AWME was 0.78 mg/mL after 24 h of incubation for all five tested phytopathogenic bacteria strains: *Pantoea agglomerans, Xanthomonas campestris*, *Pectobacterium carotovorum subsp. carotovorum, Pectobacterium atrosepticum,* and *Dickeya solani*. The minimum bactericidal concentration (MBC) ranged from 0.78 to 1.56 mg/mL against all tested strains after 24 h of incubation. The inhibition zone size of AWME (INZ) at 50 mg/mL concentration was in the range 12.2 ± 0.56 to 19.0 ± 0.28 mm, while zone size for the positive control (penicillin-streptomycin) (5000 IU/mL–5000 µg/mL) was in the scale of 20.63 ± 0.53 to 24.0 ± 0.35 mm as revealed by standard disk diffusion assay. For the first time, our findings indicated the substantial antibacterial potential of AWME of *H. illucens* larvae fat against these actual phytopathogens, thus paving the way for further research to determine the mechanism of action in crop protection.

## 1. Introduction

Bacterial phytopathogens are among the most important causal agents of plant diseases, with a negatively increased economic impact on crop production. Phytopathogens affect all food-producing plants colonizing either their surface or tissues [1]. They cause symptoms such as spots, blights, cankers, tissue rots, and/or hormone imbalances that lead to the plant overgrowth, stunting, root branching, and leaf epinasty [1,2]. Bacterial soft rot caused by pectinolytic *Pectobacterium* species (soft rot *Pectobacteriaceae*) and *Dickeya* species (*Solanum tuberosum*) is one of the major disease of the potato production in many potato-growing regions of the world [3,4]. High genotypic and phenotypic diversity of these bacteria caused disease symptoms on a wide range of host plants. *Pectobacterium* induce tissue maceration or rot symptoms in plants. These pathologies caused by the phytopathogen production of extracellular enzymes degrade cell wall constituents, such as pectate lyases and other pectinases, cellulases, and proteases [5,6]. *Xanthomonas* belongs to gram-negative bacteria that can infect such crops as beans, rice, citrus, and cotton [7]. *Xanthomonas campestris pv campestris* invades the vascular system or the mesophylls and cause black rot diseases for cruciferous crops such as mustard, cabbage, broccoli, cauliflower, brussels sprouts, and radish worldwide [8]. *Dickea solani* and *Pantoea agglomerans* distribute among various broad of host range, many crops ranging from potato to banana, fire blight disease of ornamentals, fruit trees, bushes, the soft rot diseases, and others [9].

However, the control of diseases caused by the pathogens listed above is difficult due to the limited efficacy of existed biological or chemical agents and the resistance to antibiotics long-term overuse [10]. The combination of some reliable methods was applied to control plant diseases, including mechanical (such as hot water, steam, dry hot air, solar) or UV irradiation treatment, and chemical processing by antibiotics, natural bactericides, or synthetic anti-microbial peptides [10,11]. It is worth stressing that, at present, there are no methods enable to eradicate post-infection besides the only preventive measures, which are commonly applied to limit or eliminate their further prevalence. Persistent infection increases the levels of morbidity and mortality globally and are an essential cause of plants’ recurrent infective diseases. This might also be a prime reason for the crop disease outbreaks and recurrence, even with the frequent use of antimicrobial compounds and other management methods in the field [12,13]. Antimicrobial compounds of natural origin stand up as frontiers in the attempts of the overused antibiotics replacement.

In this study, we used the black soldier fly *H. illucens* (BSFL) as a primary source to isolate compounds from their larvae fat with the antimicrobial capacity. Compared with other insects, BSFL does not accumulate pesticides or mycotoxins and have higher saturated fat content, suggesting a safe and economic prospect [14]. The BSFL demonstrated a high grade of balance between saturated and unsaturated fatty acids (FAs) with pronounced antiviral, antibacterial, and antiprotozoal activities [15]. Most antibacterial activities of BSFL larvae attributed to soluble peptide fractions isolated by acidic treatment of the whole body of larvae (WBL) [16]. The methanol extract of WBL of *L. cuprina* demonstrated the in vitro antibacterial activity of this extract against seven selected human wound pathogens (*Staphylococcus aureus, methicillin-resistant S. aureus, S. epidermidis, Streptococcus pyogenes, Klebsiella pneumoniae, Pseudomonas aeruginosa,* and *Escherichia coli*). The reconstituted larval extract was highly robust and thermally stable [17]. These observations substantiated the feasibility of the methanol extraction method in the production of larval extract. Nevertheless, so far none of the studies demonstrated the antibacterial activity of BSFL AWME of larvae fat against a wide range of phytopathogenic bacteria.

*H. illucens* larvae consist of 15% to 49% of fat, providing a rich source of lipids [18]. This study aimed to develop a new procedure to constitute the AWME composition via modified acidic water-methanol extraction and to demonstrate in vitro the antibacterial activity of this extract against five crucial plant pathogenic bacteria *Xanthomonas campestris subsp. campestris, Pantoea agglomerans*, *Dickeya solani, Pectobacterium carotovorum subsp. carotovorum,* and *Pectobacterium atrosepticum*. To our knowledge, the *H. illucens* larvae fat was directly extruded from alive BSFL, never used before as a natural source for this extraction. This study addresses the urgent needs of new antibacterial agents for agriculture crop protection.

## 2. Materials and Methods

### 2.1. BSFL Sample, Reagents, and Supply

Fat was isolated from alive *Hermetia illucens* larvae of 15 days old by using mechanical pressing machine and provided by the company NordTechSad, LLC (Arkhangelsk, Russia), and used for this study. All experiments were performed with fat from BSFL under sterile condition at room temperature.

Hydrochloric acid (HCl), methanol (CH_3_OH), carbon tetra chloride (CCl_4_), dimethylformamide (DMFA), hexane (C_6_H_14_), dichloromethane (CH_2_Cl_2_), dimethyl sulfoxide (DMSO), chloroform (CHCl_3_), formaldehyde (CH_2_O), acetonitrile (CH_3_CN), and isoamyl alcohol (C_5_H_11_OH) were purchased from Thermo Fisher Scientific, Waltham, MA, USA. Luria-Bertani broth and agar (LB), Mullar Hinton agar (MHA) were purchased from Sigma-Aldrich, St. Louis, MO, USA. Tissue culture 96-well plates (TPP, Trasadingen, Switzerland), 100-X mixture of antibiotics penicillin-streptomycin (5000 U/mL–5000 µg/mL) (Gibco, Thermo Fisher Scientific, Waltham, MA, USA), Petri dishes (90 mm) (Pertin, Saint Petersburg, Russia), paper discs, 6 mm diameter (Himedia, Mumbai, India), sterile swab (Nigbo Greetmed medical instruments CO., LTD., Nigbo, China) were used for this work.

### 2.2. Bacterial Strains and Culture Growth

Antibacterial efficacy of AWME was evaluated against the following bacterial strains: *Pantoea agglomerans (Pagg)* (ATCC 27995), *Xanthomonas campestris* (Xcc) (ATCC 13951), *Pectobacterium carotovorum subsp. carotovorum* (Pbc) (ATCC 15713), *Pectobacterium atrosepticum* (Pba) (ATCC BAA-672D) and *Dickeya solani* (Dsol) (NCBI IPO 2222). The bacterial strains used in this study were purchased from the American Type Culture Collection (ATCC), Manassas, USA.

The bacterial strains were stored in glycerol stock (40%, *v/v*) at −80 °C. To culture, they were incubated overnight in 10.0 mL of LB broth at 28 ± 0.5 °C. The overnight culture was adjusted to the 0.5 OD_600_, equal 0.5 McFarland (1–5 × 10^8^ CFU/mL). All experiments were performed under aseptic conditions (Safe Fast Elite, Ferrara, Italy).

### 2.3. BSFL Fat Extraction

The BSFL fat extract (AWME) was prepared by treatment of 3 g of larvae fat with 10 mL of mixture of water (Milli Q quality), methanol (99.9%, HPLC grade), and hydrochloric acid (37%) in the ratio (90:9:1) at pH ˂ 1. Briefly, 3 g of larvae was melted in 10 mL of extraction solution under hot tap water (52 °C) during 5.0 min, then homogenized thoroughly by vortex V-1 (BIOSAN/Latvia) for 10.0 min and subjected to extraction on the orbital shaker Mixmate Eppendorf AG, Hamburg Germany) at 2000 rpm/min for 24 h at room temperature. Next, the mixture was sonicated at 35 °C for 10.0 min (Elmasonic S 30H, Singen, Germany), then finally homogenized on ULTRA TURRAX-25 homogenizer (IKA, Deutschland, Germany) at 17,000.0 rpm/min for 10 min. The insoluble fat was separated by centrifugation at 4000× *g* for 20 min at room temperature (Centrifuge 5804, Eppendorf AG, Hamburg, Germany). The collected supernatant was used for the future experiments in this study. The extraction process was sequentially repeated three times by adding another 10 mL of AWME to the remaining fat pellet. Then, supernatants were combined together and concentrated under the vacuum (Concentrator plus, Eppendorf AG, Hamburg, Germany) at 45 °C during 13 h to obtain the 130.3 mg/mL of extracted dry substance from the larvae fat. The concentrated extract was stored at 4 °C until needed.

### 2.4. Agar Disk Diffusion Assay

The diameters of growth inhibition zone for all tested bacteria was measured according to [19]. The bacteria was incubated overnight in 10 mL of LB broth at 28 ± 0.5 °C, then adjusted to the density of 5 × 10^5^ (CFU/mL). MHA petri dishes were streaked with bacterial culture by cotton swab. Then, 50 μL of extract solution were added on 6 mm discs under sterile conditions followed by drying at ambient conditions for 30 min. As a negative control, 50 μL of the extraction reagent (AWM) was used, and as a positive control, 50 μL of antibiotics penicillin–streptomycin mixture (5000 U/mL and 5000 μg/mL/disk) was used. The discs were placed on the surface of the agar plates and incubated at 28 ± 0.5 °C for 24 h. The diameters of bacterial inhibition zones surrounded the discs were measured in 24 h. All experiments were performed twice in duplicates.

### 2.5. Turbidimetric Assay

The overnight bacteria culture was adjusted to the density of 5 × 10^6^ (CFU/mL) in fresh LB broth. AWME was diluted in LB broth to 50 mg/mL concentration. 100 µL of bacteria with 5 × 10^6^ (CFU/mL) concentration was loaded in each well of 96-well plate (TPP, Trasadingen, Switzerland). Then, the larvae fat extract was subjected to two-fold dilutions in the same plate by adding 100 µL of start extract concentration of 50 mg/mL in the first well following by serial of dilutions to get the row of relevant AWME concentrations as 25, 12.5, 6.25, 3.13, 0.78, 0.195, 0.097, and 0.00 mg/mL in the wells. The negative (100 µL of AWM extraction reagent) and positive (100 µL of penicillin-streptomycin (P/S) with 50 µg/mL concentration) controls were subjected to the two-fold dilutions as described above for AWME dilutions. Each dilution was used in triplicates. Then, the plates were sealed with a film, and incubated at 28 ± 0.5 °C with shaking at 130 rpm/min for 24 h. The optical density was measured at intervals of every 2 h from 0 h to 24 h at 600 nm by using CLARIOstar microplate reader (BMG LABTECH, Ortenberg, Germany).

### 2.6. Determination of Minimum Inhibitory Concentrations by Turbidimetric Assay

The minimum inhibitory concentrations (MICs) was determined according to [20]. The 100 µL of larvae extract with 100 mg/mL of started concentration was diluted in 96-well plates to the final concentrations of 25, 12.5, 6.25, 3.13, 0.78, 0.195, and 0.097 mg/mL through a serial of dilutions by 100 µL of tested bacteria mixture started with 5 × 10^6^ (CFU/mL) density. Similar to the fat extract, we prepared the positive control (P/S) in the same range of concentrations. The plates were incubated at 28 ± 0.5 °C with shaking 130 rpm/min for 24 h. The MICs were defined by the visual inspection as the lowest concentration of AWME that were able to inhibit the bacterial growth. The experiment was performed under sterile conditions to avoid any unwanted bacterial contaminations.

### 2.7. Determination of Half of Inhibitory Concentration (IC_50_)

The half of inhibitory concentration (IC_50_) was calculated from the Turbidimetric assay at 6 h, 12 h, and 24 h. Briefly, the 100 μL of bacterial suspension at the final density 5 × 10^5^ (CFU/mL) was added to 96-well plates, which contained an aliquot of 100 μL of BSFL fat extract previously diluted in LB broth to the final concentrations of 25, 12.5, 3.13, 1.56, 0.78, 0.39, 0.195, 0.097, and 0.00 mg/mL. Positive control (P/S) was prepared and added as mentioned above. For each sample, IC_50_ was determined using non-linear regression mode of GraphPad PRISM™ software, version of 6.07 (Graph Pad Software, Inc., San Diego, CA, USA). The IC_50_ values are expressed as the inhibitory dose of AWME that reduced the tested bacteria growth by 50% of the untreated bacteria control.

### 2.8. Determination of Minimum Bactericidal Concentration (MBC)

Minimum bactericidal concentration (MBC) for AWME and positive control (P/S) was determined according to [21,22] with a slight modification. Thus, 50 μL of the mixture from the 1× MIC (0.78 mg/mL), 2× MIC (1.56 mg/mL) and 3× MIC (3.125 mg/mL) wells of the TB assay was sub-cultured on MH agar plates after 24 h of the incubation. MH agar plates were further incubated for 48 h. The lowest concentration of the AWME extracts, at which no bacterial growth was observed, was accepted as MBC for this strain. The experiment was repeated in triplicate for each strain.

### 2.9. Gas Chromatography-Mass Spectrometry (GC-MS) Analysis

The GC-MS analysis of the BSFL AWME was conducted by using GCMS-QP2010 ultra mass spectrometer (Shimadzu, Canby, OR, USA) and PAL 5000 autosampler (Gerstel, Zwingen, Switzerland). Separation of bioactive compounds was carried out by using capillary column DB-5ms (30 m × 0.25 mm × 0.25 mm) coated with non-polar silphenylene polymer virtually equivalent polarity to 5% diphenyl and 95% dimethylpolysiloxane stationary phase (Restek, Bellefonte, PA, USA. Operation conditions were as follows: the injector and detector temperatures were set at 280 °C and 250 °C, respectively. The 1 μL of BSFL AWME solution was injected automatically by split-less mode. The temperature program was as follow: the initial temperature 40 °C was held for 1 min, and then it was increased at 15 °C/min until 210 °C, held for 0 min, increased at 5 °C/min until 216 °C, held for 0 min, increased at 40 °C/min until 300 °C, and finally held for 14.87 min. The helium was used as the carrier gas, with a linear velocity of column flow from 1 to 15 mL/min, and the column head pressure was 50.4 kPa. Fatty acids content of AWME were identified by using a mass spectrometer (MS). MS operated in the electron ionization mode and mass spectra were collected within m/z from 33 to 1000. The qualitative analysis of compounds based on the comparison of their spectral mass with those of NIST mass spectra database (NIST 08, MS v.2., MD 20899, 2008). Compound chromatogram peaks’ matching similarity index (SI) greater than 70% in NIST library were assigned.

### 2.10. Statistical Analysis

Statistical analyses were performed using the two-way ANOVA and post-hoc Tukey’s tests (*p* < 0.05). All results assessed by the standard deviation (SD) and standard error of the mean (SEM) using built-in algorithms of GraphPad Prism™ software, version 6.07 (San Diego, CA, USA).

## 3. Results

### 3.1. Extraction of Bioactive Compounds from the Hermetia illucens Larvae Fat

To choose the best solvent for the extraction of the soluble form of antimicrobial constituents, we tested the BFSL fat solubility in 21 organic solvents individually or in combination. To increase the yield of the solvent extracted chemicals, we managed the concentration of fat in the range 10% to 30% (*w/v*) for each solvent at room temperature. The fat heating under hot tap water and testing of chemicals in the mixture with H_2_O used to increase its hydrophilicity. We found that the fat was soluble in almost all non-polar reagents without a difference in its hydrophobicity (Table 1). However, we observed the formation of the light fraction on the top of double layers, when extracted by hydrophobic solvents such as CHCl_3_, DMSO, and C_6_H_14_. We found that during the dissolution of BFSL fat in hydrophilic reagents, CH_3_CN, CH_3_OH, or C_15_H_11_OH, the cloudy layer formed at the bottom of the extracted solvent. The increasing fat concentration from 10% to 30% (*w/v*) revealed the decreasing solubility of fat almost for every organic solvent, except for tetrachloromethane, pentanol, and a mixture of chloroform- dimethylformamide (9:1, *v/v*) as shown in Supplementary 3.

When we evaluated the fractions of soluble or partially soluble fat on its antimicrobial efficacy against phytopathogenic bacteria, none of them demonstrated a positive result (data not shown). Considering the empirically observed positive influence of hydrophilicity on BSFL fat solubility, we increased the content of water and methanol in extracting reagent. As the larvae fat contained free fatty acids, the presence of organic acids might catalyze the cascade of lipid hydrolysis at increased temperature by the scheme:

RCOOR′ + H_2_O ⇌ RCOOH + R′OH  (Hydrolysis) (1)

 RCOOH + R′OH ⇌ RCOOR′ + H_2_O  (Esterification) (2)

  RCOO R′ + R′′OH ⇌ RCOO R′′ + R′OH  (Alcoholises)(3)

The reactions shown in Equations (1)–(3) are the most widely used in fatty acid and lipid chemistry of conversion the acids to esters or vice versa. We optimized the BSFL fat extraction conditions and formulated its composition as a mixture of deionized water:methanol:hydrochloric acid at volume ratio 90:9:1 (%) (AWM). In our mix, the HCl plays an essential catalytic role providing a higher level of extraction of fatty acids from BSFL fat. In addition to fatty acids, these reactions produce the methyl esters synthesis, which is the starting point for the most oleochemical and glycerol production as valuable byproducts. The well-known alcoholysis or methanolysis of triacylglycerols used to prepare methyl esters; a process frequently referred to as transesterification [23]. Using AWM solvent, we were able to extract up to 4.33% of the active fatty acids and its derivatives from BSFL fat.

### 3.2. Examination of Antibacterial Activity against Phytopathogens

**Figure 1:**
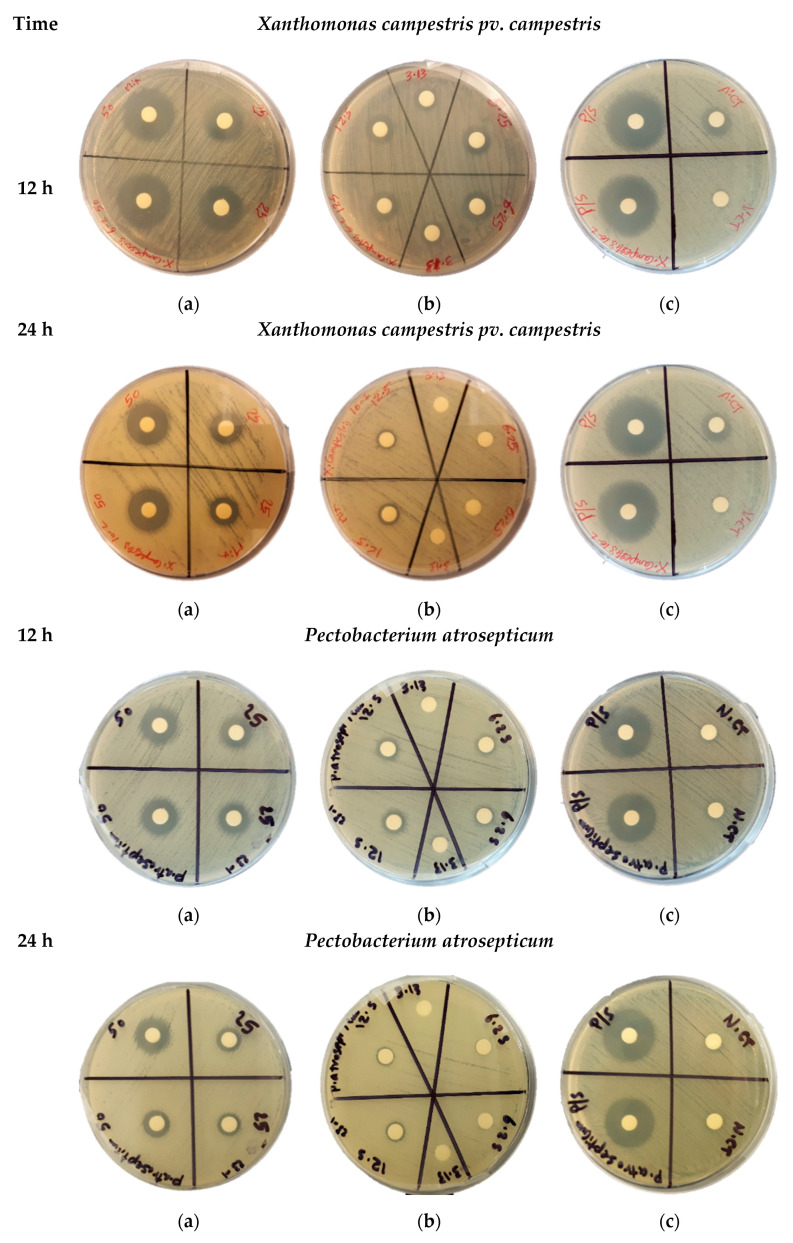
The bacterial growth inhibition assay. The representative images of zones of bacterial growth inhibition were measured after 12 h and 24 h of overnight incubation of the discs loaded on petri dishes agar with *Xanthomonas campestris pv campestris* and *Pectobacterium atrosepticum* bacteria lawn. The discs with tested samples were loaded with 50 µL of AWME from larvae fat with concentrations of 50 and 25 mg/mL (**a**); 12.5, 6.25, and 3.13 mg/mL (**b**); 50 µL of P/S samples with concentration 5000 U/mL–5000 µg/mL was used as positive control (**c**). The 50 µL of pure AWM extracting solution used as a negative control (N-CT) (**c**). All samples were loaded in duplicates.

The antibacterial effect of acidic water-methanol extract (AWME) from BSFL fat against five significant phytopathogens was measured using the agar disk diffusion assay. The results of bacterial growth inhibition of *Xanthomonas campestris pv. campestris and Pectobacterium astrosepticum* during 12 h and 24 h of treatment with different concentrations of AWME in the range 6.5 to 50 mg/mL shown in Figure 1.

AWME effectively inhibited bacterial growth for all tested Xcc, Pagg, Dsol, Pcc, and Pba pathogenic bacteria (Table 2). The AWME suppressed the growth of all phytopathogens in a dose-dependent manner in the range 50 to 3.13 mg/mL of tested concentrations (Table 2). Thus, the Pagg and Dsol strains were the most susceptible during 12 h of incubation to AWME treatment at minimal 3.13 mg/mL of its concentration. In contrast, for all other strains, the same effect was observed at 12.5 mg/mL of AWME concentration. The bacteria susceptibility to the AWME treatment decreased after 24 h of bacteria growth, compared with 12 h, as seen from Figure 1 and Supplementary 1. The IZD size after 12 h of incubation was the highest for Xcc strain (23.5 ± 035 mm) and lowest for the Pba strain (14.75 ± 0.75 mm) at 50 mg/mL of AWME concentration, while after 24 h it decreased to 19.0 ± 0.28 mm and 12.2 ± 0.56 mm, respectively. A decrease of AWME level in the range from 50 mg/mL to 3.13 mg/mL led to essential IZD size reduction during 12 h of incubation, compared with 24 h. Thus, for the most susceptible Xcc strain IZD size was decreased by 69.1% from 23.5 ± 035 mm to 7.25 ± 0.28 mm after 12 h of incubation, while after 24 h only by 57.0% from 19.0 ± 0.28 mm to 8.0 ± 0.7 mm. The slight fluctuation of IZD size between 20.63 ± 053–24.0 ± 0.35 mm for positive control penicillin/streptomycin at 5000 IU/mL to 5000 µg/mL loads did not substantially change between 12 h and 24 h of incubation. The actual IZD size due to AWME treatment demonstrated a significant difference in comparison to Positive control at **** P < 0.0001 as shown in Supplementary 1. These results indicated that chemical compounds extracted from *H. illucens* larvae fat by hydrochloric acid:water:methanol extraction possess efficient antibacterial activity against five plant pathogens.

### 3.3. The Potency of AWME from Larvae Fat on Bacteria Growth and Proliferation in Turbidimetric Assay (TB)

**Figure 2:**
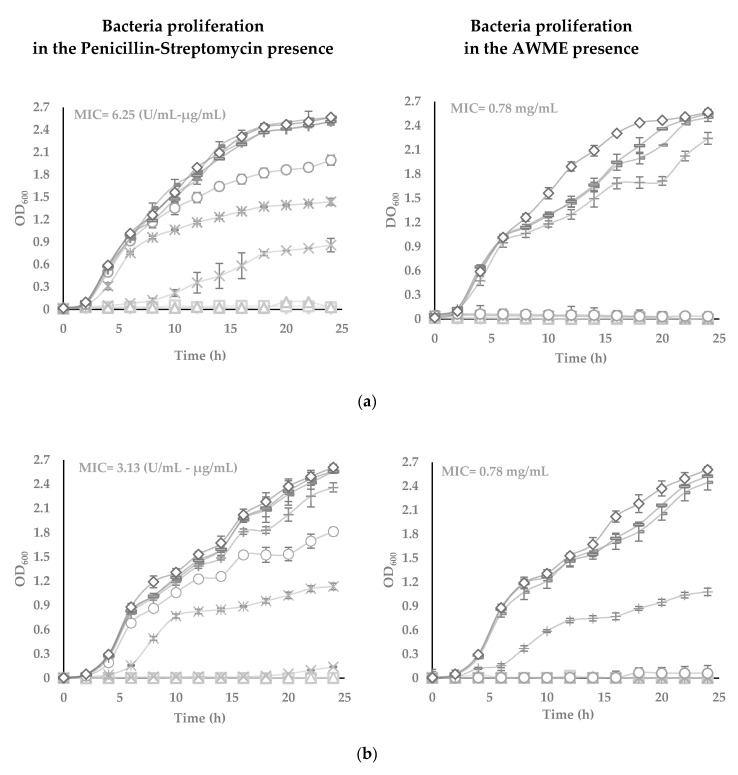

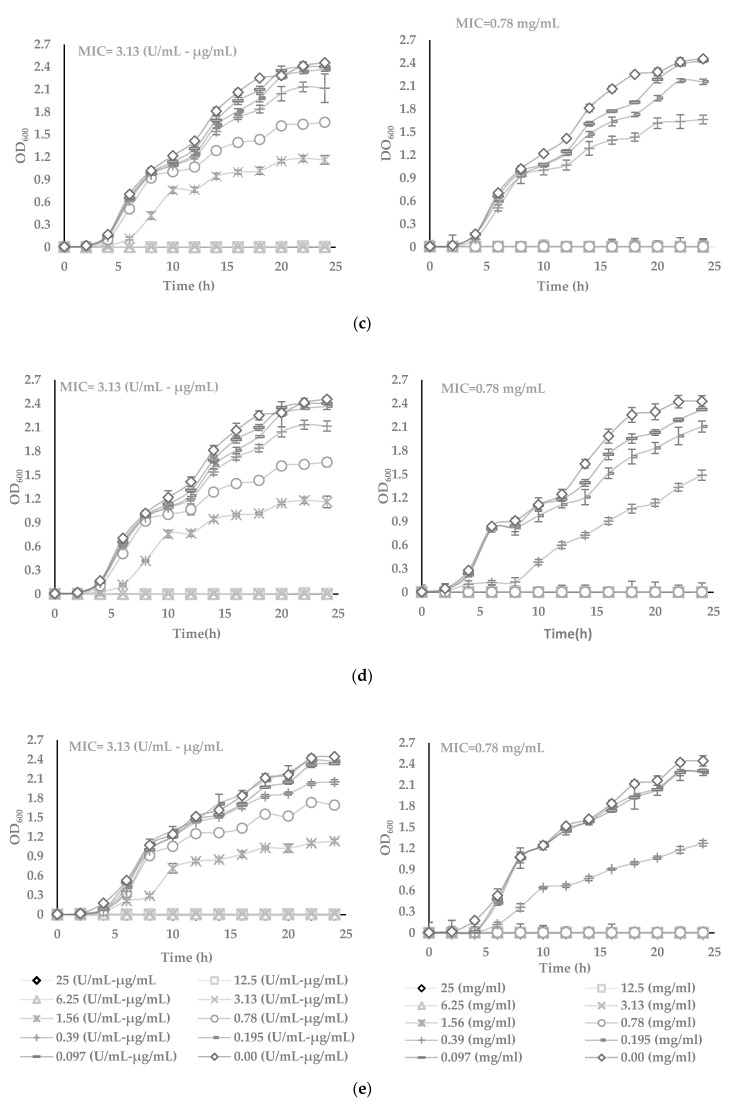
The turbidimetric assay performed in the sterile 96-well plates, in the range of 25 to 0.0097 µg/mL of AWME and P/S concentrations, in triplicates. Five bacteria strains tested: (**a**) *Xanthomonas campestris subsp. campestris;* (**b**) *Pantoea agglomerans;* (**c**) *Dickeya solani;* (**d**) *Pectobacterium carotovorum subsp. carotovorum*, and (**e**) *Pectobacterium atrosepticum.* Minimum inhibitory concentration (MIC) value against bacterial growth was determined for the wells, which appear to stop the growth and remain transparent. Each value represents the mean of three independent experiments ± SD (*n* = 3). Differences were considered significant at *p* < 0.05.

The antimicrobial effects of AWME against plant pathogenic species Xcc, Pagg, Dsol, Pcc and Pba were further evaluated using turbidimetric (TB) assay in the range from 25 to 0.097 mg/mL of AWME concentration (Figure 2). TB assay results demonstrated the increase of the AWME antimicrobial efficacy in a concentration-dependent manner, similar to the one observed from the agar disk diffusion assay (Figure 1). The measuring of the optical density of the cell suspension at 600 nm within 24 h assessed the effect of AWME concentration on bacterial proliferation. As shown in Figure 2, in the presence of 0.39 mg/mL AWME, the bacterial proliferation was retarded for all strains throughout the experiment except Xcc strain, whose proliferation recovered after 20 h of incubation. We observed the difference in the rate of proliferation among tested and control groups in the late log phase. This trend continued at the stationary phase, when the cultures reached higher 2.4 OD_600_ for the control group, while the density among AMWE-treated bacteria was in the range of 1.07 to 1.5 OD_600_. Besides, the presence of AWME at 0.39 mg/mL concentration delayed the lag phase time up to 6 h for Pagg, Pba, and Pcc strains, except Xcc (Figure 2).

The minimum inhibitory concentration (MIC) value was 0.78 mg/mL for all tested phytopathogens. In comparison, MIC for the positive control penicillin-streptomycin was 3.13 U/mL-µg/mL for all strains, except Xcc, which was 6.25 U/mL–µg/mL. At the same time, TB results showed apparent differences in the susceptibility of tested phytopathogens to the AWME treatment. The minimum bactericidal concentration (MBC), which kills 99.9% of the bacteria, indicates the antimicrobial potency of AWME. MBC of AWME was 0.78 mg/mL for all phytopathogenic strains except Pagg, which was the less susceptible to AMWE with MBC 1.56 mg/mL. MBC of the positive control (P/S) was 6.25 U/mL–µg/mL only for Xcc strain and 3.125 U/mL–µg/mL for all other strains (Figure 2). Besides, the BSFL extraction reagent (AWM reagent) further verified as vehicle control for its antibacterial capability within the same range of concentrations as used in the TB assay for AMWE. We found that AWM reagent does not affect all tested bacteria proliferation (data not shown). These results indicate the AWME potency for bacterial growth inhibition, especially of such significant pathogens as *Pantoea agglomerans*, *Dickeya solani* and *Pectobacterium atrosepticum.*

### 3.4. Determination of the 50% Inhibitory Concentration (IC_50_) of the Plant Pathogenic Bacteria

The effectiveness, as 50% inhibitory concentration (IC_50_) values of AWME against the growth of pathogenic species Xcc, Pagg, Dsol, Pcc, and Pba were determined at the 6 h (early), 12 h (middle) and 24 h (late) post-infections based on the dose-response curve data from our previous turbidimetric assay (Table 3).

The IC_50_ values indicated the highest resistance of *Xanthomonas campestris* *subsp. campestris* strain to AWME and standard positive control (P/S) treatments as early as 6 h post-infection (535.1 ± 0.16 µg/mL and 0, 1941 ± 0.15 µg/mL, respectively). Interestingly, those IC_50_ values of AWME and P/S for this strain retained the highest until the 24 h post-infection compared to all tested strains (485.4 ± 0.4 µg/mL and 1.893 ± 0.36 U/mL–µg/mL, respectively). On the other hand, the AWME showed the highest activity at 6 h and retained the highest during 24 h of monitoring against Pba and Pagg strains with IC_50_ values equal to 290.3 ± 0.07 vs. 400.2 ± 0.35 and 299.5 ± 0.12 vs. 366.8 ± 0.38 µg/mL, respectively, as shown in Supplementary 2. In addition, the AWME showed high stability and the ability to maintain the consistent responsiveness throughout 24 h of *Dickeya solani* strain treatment as seen by the similar IC_50_ values (451.8 ± 0.1, 455.1 ± 0.2 and 441.8 ± 0.36 µg/mL at 6 h, 12 h, 24 h, respectively). In contrast, all recorded IC_50_ values of the standard positive control have fluctuated within all tested strains. These results demonstrate that proposed AWME from BSFL fat can prevent and sustainably inhibit the proliferation and growth of the plant pathogens as early as 6 h of incubation, thus having the potential to be used as an antibacterial agent.

### 3.5. Gas Chromatography-Mass Spectrometry (GC-MS) Analysis of AWME

**Figure 3:**
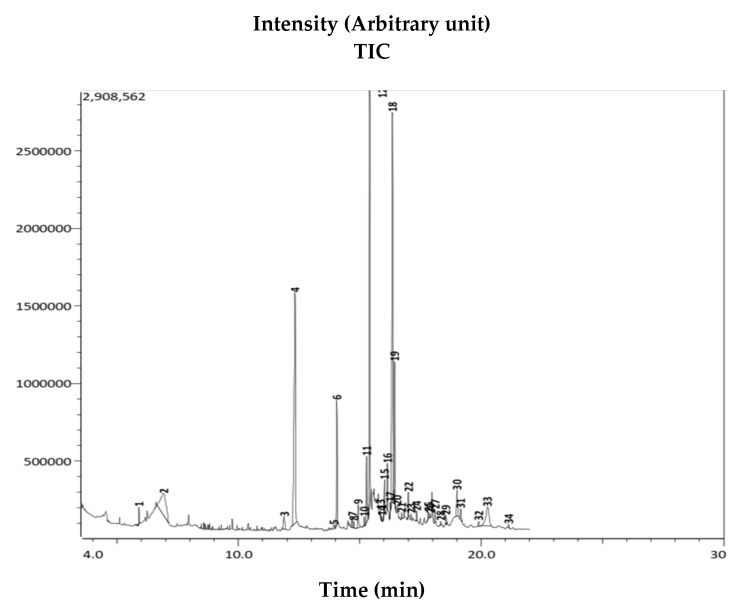
Gas Chromatography-Mass Spectrometry (GC-MS) chromatogram of AWME from BSFL fat. The chemical identity of 34 compounds from AWME fat detected by GC-MS was determined based on NIST Library of chemical compounds.

The GC-MS analysis identified 34 organic compounds in the AWME of larvae fat (Figure 3). The chemical profile of these compounds was determined based on the National Institute of Standards and Technology (NIST, USA) database. After comparing the mass spectrum of the unknown AWME components with the range of the known chemicals from NIST library, the similarity of GC-MS spectrums more than 70% was considered as the main criteria for that selection.

Among 13 dominant (>1%) organic compounds, the three most abundant represented more than 50% of the content, i.e., 22.22% of octadec-9-eonic acid (18:1, syn. oleic acid), 20.34% of n-hexadecanoic acid (16:0, syn. palmetic acid), and 18.48% of dodecanoic acid (12:0, syn. lauric acid) (Table 4). The other compounds were presented in trace amount (less than 1%), but possessed interesting biological activity and remained as subjects of our further study. Only the highly presented extract (≥1%) compounds, or the ones characterized in the literature by antimicrobial capacity, were chosen for the final analysis (Table 5).

## 4. Discussion

The Black Soldier Fly (BSF) *H. illucens* is a valuable natural resource of biologically active compounds and one of the richest among other insects in the lipids, which can reach up to 45% depending on the rearing source [24]. The composition of lipids varies depending on the method of larvae processing that can yield various fatty acids (FAs) profile [25]. The larvae’s lipid profile is mainly rich in lauric, myristic, palmitic, oleic, capric, linoleic, and other medium-chain fatty acids. Thus, myrestic acid has a broad spectrum of antibacterial effects [26], larvaecidal and repellent activities [27]. Choi and Jiang [28] reported the activity of hexanedioic acid extracted from BSFL against Gram-positive and Gram-negative bacteria. Makkar et al., 2014 [15] reported a high nutritional value of unsaturated oleic (18:1, n-9) and linoleic acids (18:2, n-6) from BSFL. Besides, BSFL reaches in medium-chain lauric acid with known antimicrobial activity through the disruption of the cell membrane [29]. Lauric acid and its phenolic compounds derivatives have a proven record of antimicrobial activity against lipid-coated viruses such as HIV and measles, E. coliform and Clostridium bacteria, and pathogenic protozoans such as Coccidiosis [30,31].

In this study, for the first time, we used the *H. illucens* fat manufactured through the direct pressing of the alive fly larvae as a source for FAs isolation and further characterization of AWME antimicrobial activity. In general, the low pH facilitates and enriches the FAs extraction, and this approach was successfully used for BSFL lipid extraction from the aqueous layer with different organic acids [32]. We developed a new composition of the extraction solution as a combination of water-methanol-hydrochloric acid in the ratio 90:9:1 (*v/v*), respectively. Using this solution, we were able to extract 4.43% of FAs from BSFL fat, compared to 0.52% extracted by water [33] and 2% extracted by methanol [28]. The FAs profile identified by GC-MS analysis, and presented in Table 4. In larvae fat extract, it was found 22.22% oleic and 3.02% palmetoleic monounsaturated FAs, also 20.34% palmitic, 18.48% lauric, 5.59% myristic, 5.34% stearic mono saturated FAs with the similarity index 96%, 97%, 97%, and 94%, respectively. The poly saturated fatty acid (e.g., eicosanoic acid (arachidonic acid) and poly unsaturated fatty acids (e.g., cis, cis-9, 12-Octadecadienoic acid (Z, Z) (linoleic acid)) were present in trace amount, 0.36% and 0.23%, respectively.

The more abundant amount of the saturated fatty acids compared to unsaturated identified in AWME was consistent with results published by Ushakova et al. [34]. They supposed that fatty acids in BSFL serve as energy storage. The amount of saturated fatty acids prevailed because these chemicals are less subjected to oxidation than unsaturated fatty acids. Our GC-MS analysis indicates the most abundant constituents of AWME, that confirmed the major FAs were saturated FAs and ordered in percentage scale, as shown in Table 4. Ewald et al. [35] reported, that from total amount of fatty acids in the larvae the major present filled up by lauric (C12:0), palmitic (C16:0) and oleic (C18:1 n-9) acids (52%, 12–22%, and 10–25%, respectively). Our study revealed the much higher fatty acids profile and it was shown, based on our method of extraction, that we were able to extract from BSFL larvae fat 22.22% of the oleic (C18:1 n-9), 20.34%, of palmitic (C16:0) and 18.48% of lauric acid (C12:0) (Table 4). Although several reports [36,37,38,39] showed the different amount of lauric acid, palmitic and oleic acid, it is generally accepted that the variable percentages of FAs mainly depend on the sort of insects rearing substrate.

The increasing interest in the antimicrobial effects of FAs was mainly due to emergency of antibiotic resistance problem and urgent need to develop new classes of antibacterial agents that work against novel molecular targets [40]. Antimicrobial lipids, particularly single-chain amphiphilic lipids that destabilize bacterial cell membranes, are attractive candidates to become the next-generation antibacterial agents for the bacterial infections treatment. Phospholipids, as an example of amphiphilic molecules, are the main components of biological membranes. The amphiphilic nature of these molecules defines the way in which they form the cell membranes. They arrange themselves into bilayer, positioning their polar groups towards the surrounding aqueous medium, and their lipophilic chains towards the inside of the bilayer, defining a non-polar region between two polar ones.

On the other hand, the awareness to reduce the use of the chemical pesticides by developing the alternative strategies or technologies to improve plant disease resistance and control of pathogens is highly promoted. There was growing interest in the research focused on the alternative pesticides and antimicrobial active compounds, including the plant extracts and essential oils of aromatic plants [41]. Under certain conditions, de novo mutations and selection can develop in pathogenic bacteria during one minute to hours, leading to the resistance genes arising, resulting in bacteria resistance to a single antibiotic [42]. Discovered FAs play a very crucial role in increasing resistance of plants to phytophathogens. Thus, linoleic acid found to induce systemic resistance of tobacco against the bacterial soft rot pathogen caused by *Pectobacterium* carotovorum *subsp. carotovorum* (Pcc) [43]. The lauric acid (LA) possessed the most potent activity to inhibit the growth of Gram-positive bacteria. Its monoglyceride derivative (glycerol monolaurate, (GML)) exhibited even more vigorous inhibitory activity than LA [44]. Importantly, both LA and GML are abundant in nature. They are recognized as safe by the Food and Drug Administration (FDA) in USA and exhibited full anti-infective applications, including several applications such as in agriculture [45]. The linoleic acids were phytotoxic and effective in inducing systemic resistance. In contrast, oleic acid was the least phytotoxic and caused no systemic resistance [46]. According to Blechert et al. [47], the octadecanoic acid (stearic acid) derivative, octadecatrienoic acid, (Z, Z)-methyl ester play an important role in plant defense mechanism. The plant contains linolec acid eliciting induced systemic resistance against phytopathogens. When a primary wound occurs in the plant, oligo-galacturonides signals formed, which activate octadecanoate to produce jasmonic acid, and which finally leads to the activation of the defense gene [48]. The hexadecanoic acid ethyl ester acts as antioxidant, nematicide, and pesticide. Furthermore, it may contribute to the antimicrobial and antioxidant activities [49]. As demonstrated in the present study, AWME of *H. illucens* larvae fat contains oleic, palmitic, lauric, myristic, stearic, and palmitoleic acid, which are saturated and unsaturated FAs having significant inhibition effect against bacteria that are in consistent with the activity of FAs, reported by other authors (Table 5).

FAs with the cis-form stereochemistry, mainly unsaturated fatty acids exhibit higher antibacterial activity than the corresponding trans-isomers [63]. Poly saturated FAs (eicosanoic acid) and poly unsaturated FAs such as 9, 12-Octadecadienoic acid (Z, Z)-(linoleic acid) possess a broad spectrum of antibacterial and antifungal effects [58,59,62]. Besides, the double bonds in free FAs typically have a cis-orientation showing the higher antibacterial activity than free FAs with double bonds in trans-orientation [64]. The esters of FAs, such as 9-octadecenoic acid (Z)-, methyl ester (oleic acid methyl ester), dodecanoic acid 2, 3-dihydroxypropyl ester (monolaurin), hexadecanoic acid 2-hydroxy-1-(hydroxymethyl) ethyl ester, hexadecanoic acid methyl ester (palmitic acid methyl ester) and octadecanoic acid methyl ester (stearic acid methyl ester), reported to have the sustain antibacterial effect—and even more effective than FAs [55,56,57,60,61].

In our study, the AWME from BSFL fat demonstrated antimicrobial efficacy against all five tested phytopathogens. The antibacterial effect against Xcc measured by the diameter of the inhibition zone (IZD) and formed 19.5 ± 0.28 mm, MIC was 0.78 mg/mL (as indicated in Table 2 and Figure 2). The control bacteria exhibited a lag phase for 4 h, and then OD_600_ values were rapidly increased. Nonetheless, the OD_600_ values of the AWME treatment groups showed a slight increase around 0.39 mg/mL of bacterial concentration during 4 h, or 6 h of incubation within all range of added AWME at 0.78, 1.56, 3.13, 6.25, 12.5, and 25 mg/mL (Figure 2). This finding is clear indication of AWME antibacterial activity against plant pathogenic bacteria, although we noted a slight difference in the ability to inhibit bacterial growth depending on the type of bacteria.

Of note, extracted antimicrobial peptides (AMPs) from BSFL showed IZD lower size (15.0 mm) and MIC higher (50.0 mg/mL) [16] in zone inhibition assay for Xcc during 24 h compared to our results of AWME antibacterial activity study. AMPs demonstrated an inhibitory effect against Gram-negative bacteria by IZD ranging from 9 to 13 mm, and the IC_50_ also fluctuating between 33.47 to 39.28 mg/mL after 24 h of incubation [33]. Sledz et al. [22] demonstrated the MIC and MBC values of caffeine against Xcc, Dsol, Pcc and Pba in the range 1.74 to 3.66 mg/mL and 15 to 20 mg/mL, respectively. Our data, in comparison to these results, indicated AWME from BSFL fat to be more effective against phytopathogenic bacteria in terms of IZD size in the range 12.5 ± 0.7 to 19.5 ± 0.28 mm, MIC in the range 0.87 to 1.56 mg/mL, MBC in the range 0.78 to 1.56 mg/mL and IC_50_ in the range 366.8 ± 0.38 to 485.4 ± 0.4 mg/mL after 24 h of incubation. Thus, while the AWME was more easily and more effectively isolated from BSFL fat, besides listed above superior antimicrobial characteristics, the AWME seems to be more effective than AMPs, and caffeine against actual phytopathogens.

Antibacterial efficacy of AWME was evaluated by disc assay, minimum inhibition concentration (MIC) and minimum bactericidal concentration (MBC) values. AWME showed high potency against phytopathogenic bacteria growth on both Mullar Hinton solid agar and in 96-well culture. The MBC value of AWME was determined by transferring 50 µL aliquots from the 96-well plate with MIC bacterial suspension on the Petri dish agar. The MBC of AWME was 0.78 mg/mL for all tested strains except *Pantoea agglomerans,* which was inhibited and killed at 1.56 mg/mL of AWME after 48 h of incubation at 28 °C on petri dish plates. All the above-mentioned results prove the bactericidal capacity of AWME with high potency towards all tested phytopathogens. Of note, although the MICs of larval extract in our experiments (0.78 mg/mL/well) were higher than the MICs of standard antibiotic (3.13 U/mL–6.25 µg/mL/well), the P/S antibiotic was composed of purified active ingredients, compared to the crude extracts of fly larvae [65]. Meziani et al. [66] reported that the IC_50_ for acetone extract of carob leaf was 1.5 mg/mL against *Pectobacterium atrosepticum* (Pba), while in our study IC_50_ for AWME was in the range 290.3 ± 0.07 to 400.2 ± 0.35 µg/mL at 6 h and 24 h of incubation, respectively. Although MIC for lacaic acid-D- methyl ester, extracted from an *Aloe vera,* was 93.75 μg/mL against *Xanthomonas campestris subsp. campestris* (Xcc), but the MBC was 1.5 mg/mL and besides, it was not effective against *Pectobacterium carotovorum subsp. Carotovorum* (Pcc), compared to our findings [67]. Soberón et al. [68] declared that methanolic and the aqueous extract of *Ligaria cuneifolia* and *Jodina rhombifolia* leaf have the MICs ranging from 2.5 to 156 µg/mL and 5 mg/mL, respectively, against Xcc, while these extracts were bacteriostatic. Hong et al. [69] found that phytochemicals extracted from *Zingiber officinale* rose have the MIC equal to 1.94 mg/mL against *Pantoea agglomerans.* In agreement with these data, the saturated and unsaturated fatty acids as major constituents of AWME from the *H. illuscens* larvae fat, possess the high antimicrobial effect against five important plant bacteria pathogens having MIC = 0.78 mg/mL, MBC = 0.78–1.56 mg/mL, and IC_50_ = 366.8 ± 0.38–485.4 ± 0.4 mg/mL.

The cell wall of Gram-positive bacteria consists only of a single thick peptidoglycan layer. In contrast, Gram-negative bacteria have a thin peptidoglycan layer. The lipoprotein, lipopolysaccharide, and phospholipids are forming their outer membrane layer. In this respect, the interaction of bacteria and fatty acids, the dominant constituents of the AWME of *H. illucens* larvae fat, can account for the differences in the susceptibility of Gram-positive and Gram- negative bacteria to larval extract. Such distinction may be associated with the inactivation of cell-signaling pathways, and/or degradation of an intracellular metabolic mechanism caused by the interaction of bacteria and fatty acids. Fatty acids have been reported to inhibit bacterial growth by disrupting bacterial membranes or inhibition of fatty acid synthesis [70]. Furthermore, the cis-bonds in unsaturated FAs cause a kink in the carbon chain that prevents these FAs from packing tightly into the membrane. Thus, when medium- and long-chains of unsaturated FAs are inserted into the membrane leading to the membrane’s fluidity increase, they cause cell membrane instability and disruption development [71]. Zheng et al. [72] reported the inhibition of bacterial growth by long-chain unsaturated fatty acids (LCUFA) such as oleic acid, linoleic acid, palmitoleic acid, and arachidonic acid. The target for LCUFA was the bacterial enoyl-acyl carrier protein reductase (FabI), an essential component of bacterial fatty acids synthesis.

In our future study, we will focus on finding the mechanism of action of AWME against gram-negative and gram-positive bacteria. Besides, we plan to identify the active ingredients of AWME derived from *H. illucens* larvae fat and to develop their formulations suitable for practical use in agriculture.

## 5. Conclusions

In summary, the fatty acids and its derivatives of AWME of *H. illucens* larvae fat dose-dependently inhibited the growth and proliferation of plant pathogenic Gram-negative bacteria such as *Xanthomonas campestris subsp. Campestris Pantoea agglomerans, Dickeya solani Pectobacterium carotovorum subsp. Carotovorum,* and *Pectobacterium atrosepticum*. The present study also revealed the potential of *H. illucens* larvae fat to be used for future development of novel and effective natural disinfectant(s) and antibacterial agent(s). Our data indicate the perspectives of use the *H. illucens* larvae fat extract as a novel antibacterial agent candidate composition for agriculture crop protection.

## Figures and Tables

**Table 1:** Assessment of BSFL fat solubility. BSFL fat solubilized in various single organic solvents or a combination of two. To increase hydrophilicity, the organic reagents diluted in 10-fold with H2O. The solubility of BSFL fat tested in the range 10% to 30% concentration. Solubility marks: (+) soluble; (±) partially soluble; (- - -) insoluble.

BSFL Dilution Solvent	10% Fat +H_2_O		15% Fat +H_2_O		20% Fat +H_2_O		25% Fat +H_2_O		30% Fat +H_2_O	
CCl_4_	+	±	+	±	+	±	+	- - -	+	- - -
C_6_H_14_	+	+	+	+	+	±	+	- - -	±	- - -
CH_2_Cl_2_	+	±	+	+	+	+	±	- - -	±	- - -
DMSO	±	+	±	+	±	+	±	- - -	- - -	- - -
CHCl_3_	+	- - -	±	±	±	±	±	- - -	±	- - -
DMFA	+	±	±	±	±	±	±	- - -	±	- - -
CH_3_CN	+	±	±	±	±	±	±	- - -	±	- - -
CH_3_OH	±	±	- - -	- - -	- - -	- - -	- - -	- - -	- - -	- - -
C_5_H_11_OH	+	±	+	±	+	±	+	- - -	+	- - -
C_2_H_5_OH	±	+	±	±	±	±	±	- - -	±	- - -
C_3_H_6_O	±	+	±	+	±	+	±	- - -	±	- - -
CHCl_3_ + DMFA (9:1, *v/v*)	+	±	+	±	+	±	+	- - -	+	- - -
C_6_H_14_ + C_3_H_6_O (9:1, *v/v*)	+	±	+	±	+	±	±	- - -	±	- - -
C_6_H_14_ + CH_3_CN (9:1, *v/v*)	+	±	+	±	+	±	±	- - -	±	- - -
DMSO + C_3_H_6_O (9:1, *v/v*)	+	+	±	+	±	±	±	- - -	±	- - -
DMSO + CH_3_CN (9:1, *v/v*)	+	+	±	+	±	+	±	- - -	±	- - -
CHCl3 + CH3CN (9:1, *v/v*)	+	±	+	±	+	±	±	- - -	±	- - -
CHCl_3_ + C_3_H_6_O (9:1, *v/v*)	+	+	±	±	±	±	±	- - -	±	- - -
C_5_H_11_OH + C_3_H_6_O (9:1, *v/v*)	+	±	+	±	±	±	±	- - -	±	- - -
C_5_H_11_OH + DMSO (9:1, *v/v*)	±	±	±	±	±	±	- - -	- - -	- - -	- - -

**Table 2:** Agar disk diffusion assay. Antibacterial activity was measured using the agar disk diffusion assay against *Xanthomonas campestris pv. campestris* (Xcc), *Pantoea agglomerans* (Pagg), *Dickeya solani* (Dsol), *Pectobacterium carotovorum subsp. carotovorum* (Pcc), and *Pectobacterium atrosepticum* (Pba) bacteria strains. The bacteria were exposed to 50, 25, 12.5, 6.25, and 3.13 mg/mL of extract from BSFL fat. The zone of bacterial growth inhibition was measured after 12 h and 24 h of incubation at 28 ± 0.5 °C by the diameter of clear zone surrounding of the discs (in mm). Penicillin-streptomycin (p/s) was used as an antibacterial positive control. The AWME solution was used as negative control. The results presented as means of inhibitory zone ± standard deviation of three independent experiments in duplicates. ND, not detectable; AWME, extract from BSFL fat; AWM extraction solution: water:methanol:hydrochloric acid (90:9:1, *v/v*).

	Xcc		Pagg		Dsol		Pcc		Pba	
AWME	12 h	24 h	12 h	24 h	12 h	24 h	12 h	24 h	12 h	24 h
50	23.5 ± 035	19.0 ± 0.28	15.4 ± 0.28	12.5 ± 0.7	18.5 ± 0.7	14.0 ± 0.7	20.25 ± 1.76	14.75 ± 0.35	14.75 ± 0.75	12.2 ± 0.56
25	16.5 ± 0.42	12.5 ± 0.7	10.5 ± 0.7	8.5 ± 0.35	14.2 ± 0.28	11.0 ± 0.7	13.25 ± 0.35	10.12 ± 0.18	12.25 ± 0.35	9.5 ± 0.7
12.5	12.0 ± 0.35	8.0 ± 0.7	9.3 ± 0.21	7.0 ± 0.0	11.0 ± 0.7	9.0 ± 0.7	8.25 ± 0.35	7.0 ± 0.0	9.12 ± 0.18	7.12 ± 0.18
6.25	7.25 ± 0.28	ND	8.0 ± 0.7	ND	9.0 ± 0.7	7.0 ± 0.0	7.25 ± 0.35	ND	7.37 ± 0.18	ND
3.13	ND	ND	7.0 ± 0.0	ND	7.0 ± 0.0	ND	ND	ND	ND	ND
P/S	23.75 ± 0.35	24.0 ± 0.35	20.75 ± 0.35	21.0 ± 0.7	23.75 ± 0.7	23.75 ± 0.7	21.12 ± 0.18	21.0 ± 0.7	21.0 ± 0.7	20.63 ± 053
AWM	7.0 ± 0.0	ND	ND	ND	ND	ND	ND	ND	ND	ND

**Table 3:** IC_50_ analysis for phytopathogens treated with AWME from fat vs. positive control (penicillin/streptomycin) for 6 h, 12 h, and 24 h.

	IC_50_ (µg/mL) of AWME against Treated Bacteria			IC_50_ (µg/mL) of Positive Control against Treated Bacteria		
Bacteria species	6 h	12 h	24 h	6 h	12 h	24 h
Xcc	535.1 ± 0.16	477 ± 0.25	485.4 ± 0.4	1.941 ± 0.15	1.844 ± 0.26	1.893 ± 0.36
Pagg	299.5 ± 0.12	383.6 ± 0.22	366.8 ± 0.38	1.103 ± 0.13	1.638 ± 0.22	1.283 ± 0.37
Dsol	451.8 ± 0.07	455.1 ± 0.2	441.8 ± 0.36	1.060 ± 0.1	1.675 ± 0.19	1.306 ± 0.35
Pcc	317.6 ± 0.07	386.6 ± 0.22	431 ± 0.35	1.099 ± 0.13	1.782 ± 0.19	1.412 ± 0.35
Pba	290.3 ± 0.07	376.1 ± 0.22	400.2 ± 0.35	1.088 ± 0.07	1.630 ± 0.22	1.326 ± 0.35

**Table 4:** Chemical content of AWME from BSFL fat.

Peak Number	Retention Time	Content (%)	Compound Name (NIST Library)	Chemical Formula	Molecular Weight (g/Mol)	**Similarity (%)**
1	5.9	0.61	1,2-Propanediol, 3-chloro	C_3_H_7_ClO_2_	110	93
2	6.92	6.88	1,2,3-Propantriol	C_3_H_8_O_3_	92	97
3	11.888	1.02	beta.-D-Glucopyranose, 1,6-anhydro	C_6_H_10_O_5_	162	92
4	12.335	18.48	Dodecanoic acid (lauric acid)	C_12_H_24_O_2_	200	97
5	13.335	0.3	2,4-Dodecadienal, (E,E)- (aromatic substance)	C_12_H_20_O	180	80
6	14.059	5.59	Tetradecanoic acid (myristic acid)	C_14_H_28_O_2_	228	97
7	14.682	0.41	Dodecanoic acid, ethenyl ester (lauric acid vinyl ester	C_14_H_26_O_2_	226	81
8	14.753	0.24	Pentadecanoic acid (saturated fatty acid)	C_15_H_30_O_2_	242	83
9	14.937	1.17	3-Cyclopentylpropionic acid, 2-dimethylaminoethyl ester	C_12_H_23_NO_2_	213	91
10	15.186	0.35	Hexadecanoic acid methyl ester (palmetic acid methyl ester)	C_17_H_34_O_2_	270	88
11	15.286	3.02	cis-9-Hexadecenoic acid (palmetoleic acid)	C_16_H_30_O_2_	254	96
12	15.412	20.34	n-Hexadecanoic acid (palmetic acid)	C_16_H_32_O_2_	256	96
13	15.858	0.18	Dodecanoic acid, 2-hydroxy-1-(hydroxymethyl) ethyl ester	C_15_H_30_O_4_	274	73
14	15.918	0.14	Hexadecanoic acid	C_16_H_32_O_2_	256	78
15	16.02	1.32	Dodecanoic acid, 2,3-dihydroxypropyl ester (monolaurin)	C_15_H_30_O_4_	274	83
16	16.138	1.62	9-Octadecenoic acid (Z)-, methyl ester (oleic acid methyl ester)	C_19_H_36_O_2_	296	87
17	16.257	0.34	Octadecanoic acid, methyl ester (stearic acid methyl ester)	C_19_H_38_O_2_	298	86
18	16.347	22.22	Octadec-9-eonic acid (oleic acid	C_18_H_34_O_2_	282	95
19	16.437	5.34	Octadecanoic acid (stearic acid)	C_18_H_36_O_2_	284	94
20	16.537	0.27	Linoleic acid ethyl ester	C_20_H_36_O_2_	308	75
21	16.721	0.23	9,12-Octadecadienoic acid (Z,Z)-(linoleic acid)	C_18_H_32_O_2_	280	89
22	17.002	0.97	Fumaric acid, 2-dimethylaminoethyl heptadecyl ester	C_25_H_47_NO_4_	425	82
23	17.114	0.24	Tetradecanoic acid, 2-hydroxy-1-(hydroxymethyl) ethyl ester	C_17_H_34_O_4_	304	86
24	17.346	0.36	Eicosanoic acid (Arachidic acid)	C_20_H_40_O_2_	312	92
25	17.821	0.35	Octanoic acid, 2-dimethylaminoethyl ester	C_12_H_25_NO_2_	215	87
26	17.902	0.15	cis-9-Hexadecenal	C_16_H_30_O	238	83
27	18.1	0.51	Hexadecanoic acid, 2-hydroxy-1-(hydroxymethyl) ethyl ester	C_19_H_38_O_4_	330	94
28	18.336	0.15	9-Octadecanoic acid (Z)-	C_18_H_34_O_2_	282	83
29	18.551	0.33	Tetradecanamide	C_14_H_29_NO	227	91
30	19.011	1.45	9-Octadecenoic acid, 1,2,3-propanetriyl ester, (E,E,E)-	C_57_H_104_O_6_	884	93
31	19.162	0.68	Oleoyl chloride	C_18_H_33_ClO	300	90
32	19.905	0.22	Octadecanamide	C_18_H_37_NO	283	92
33	20.262	3.52	Dodecanoic acid, 1,2,3-propanetriyl ester	C_39_H7_4_O_6_	638	85
34	21.134	0.2	Cholesterol, pentafluoropropionate	C_30_H_45_F_5_O_2_	532	88

**Table 5:** The content and published biological activity of major constituents of AWME of *H. illucens* larvae fat.

Name of Compounds	Content (%)	Biological Activity
Octadec-9-eonic acid (oleic acid)	22.22	Antibacterial [50]
n-Hexadecanoic acid (palmitic acid)	20.34	Antimicrobial [51]
Dodecanoic acid (lauric acid)	18.48	Antibacterial [52]
Tetradecanoic acid (myristic acid)	5.59	Antibacterial [26]
Octadecanoic acid (stearic acid)	5.34	Antimicrobial [53]
cis-9-Hexadecenoic acid (palmitoleic acid)	3.02	Antibacterial [54]
1,2,3-Propantriol Hexadecanoic acid, 2-hydroxy-1-(hydroxymethyl) ethyl ester cis-9-Hexadecenal	6.88 0.51 0.15	Antimicrobial and antiseptic [55] Antimicrobial [55] Antimicrobial [55]
9-Octadecenoic acid (Z)-, methyl ester (oleic acid methyl ester)	1.62	Antimicrobial [56]
Dodecanoic acid, 2,3-dihydroxypropyl ester (monolaurin)	1.32	Antimicrobial [57]
Eicosanoic acid (arachidic acid)	0.36	Antibacterial, antifungal, antioxidant [58,59]
Hexadecanoic acid methyl ester (palmitic acid methyl ester)	0.35	Antibacterial and antifungal [60]
Octadecanoic acid, methyl ester (stearic acid methyl ester)	0.34	Antimicrobial [61]
9,12-Octadecadienoic acid (Z, Z)-(linoleic acid)	0.23	Antibacterial [62]

## Supplementary Materials

The following are available online at https://www.mdpi.com/2076-2607/8/9/1423/s1.

## References

[1] Kannan V., Bastas K., Devi R., Kannan V., Bastas K., Devi R. (2015). Scientific and Economic Impact of Plant Pathogenic Bacteria. Sustainable Approaches to Controlling Plant Pathogenic Bacteria.

[2] Strange R.N., Scott P.R. (2005). Plant Disease: A Threat to Global Food Security. Annu. Rev. Phytopathol..

[3] Czajkowski R., Pérombelon M.C.M., Jafra S., Lojkowska E., Potrykus M., Van Der Wolf J.M., Sledz W. (2015). Detection, identification and differentiation of Pectobacterium and Dickeya species causing potato blackleg and tuber soft rot: A review. Ann. Appl. Biol..

[4] Pritchard L., Glover R.H., Humphris S., Elphinstone J.G., Toth I.K. (2016). Genomics and taxonomy in diagnostics for food security: Soft-rotting enterobacterial plant pathogens. Anal. Methods.

[5] Anajjar B., Aitmhand R., Timinouni M., Ennaji M.M. (2007). Characterization by PCR of two strains of Erwinia carotovora isolated from the potato rhizosphere in the region of greater Casablanca Casablanca in Morocco. EPPO Bull..

[6] Cui Y., Chatterjee A., Yang H., Chatterjee A.K. (2008). Regulatory network controlling extracellular proteins in Erwinia carotovora subsp. carotovora: FlhDC, the master regulator of flagellar genes, activates rsmB regulatory RNA production by affecting gacA and hexA (lrhA) expression. J. Bacteriol..

[7] Schwartz A.R., Potnis N., Timilsina S., Wilson M., Patané J., Martins J., Minsavage G.V., Dahlbeck D., Akhunova A., Almeida N. (2015). Phylogenomics of Xanthomonas field strains infecting pepper and tomato reveals diversity in effector repertoires and identifies determinants of host specificity. Front. Microbiol..

[8] Wulff E.G., Mguni C.M., Mortensen C.N., Keswani C.L., Hockenhull J. (2002). Biological control of black rot (Xanthomonas campestris pv. campestris) of brassicas with an antagonistic strain of Bacillus subtilis in Zimbabwe. Eur. J. Plant Pathol..

[9] Motyka A., Zoledowska S., Sledz W., Lojkowska E. (2017). Molecular methods as tools to control plant diseases caused by Dickeya and Pectobacterium spp: A minireview. New Biotechnol..

[10] Czajkowski R., Pérombelon M.C.M., Van Veen J.A., Van der Wolf J.M. (2011). Control of blackleg and tuber soft rot of potato caused by Pectobacterium and Dickeya species: A review. Plant Pathol..

[11] Zimnoch-Guzowska E., Lojkowska M., Razdan M.K., Mattoo A.K. (2005). Perombelon Resistance to Bacterial Pathogens. Genetic Improvement of Solanaceous Crops Volume I: Potato.

[12] Aćimović S.G., Zeng Q., McGhee G.C., Sundin G.W., Wise J.C. (2015). Control of fire blight (Erwinia amylovora) on apple trees with trunk-injected plant resistance inducers and antibiotics and assessment of induction of pathogenesis-related protein genes. Front. Plant Sci..

[13] Hippler F.W.R., Boaretto R.M., Dovis V.L., Quaggio J.A., Azevedo R.A., Mattos-Jr D. (2018). Oxidative stress induced by Cu nutritional disorders in Citrus depends on nitrogen and calcium availability. Sci. Rep..

[14] Purschke B., Scheibelberger R., Axmann S., Adler A., Jäger H. (2017). Impact of substrate contamination with mycotoxins, heavy metals and pesticides on the growth performance and composition of black soldier fly larvae (Hermetia illucens) for use in the feed and food value chain. Food Addit. Contam. Part A Chem. Anal. Control. Expo. Risk Assess.

[15] Makkar H.P.S., Tran G., Heuzé V., Ankers P. (2014). State-of-the-art on use of insects as animal feed. Anim. Feed Sci. Technol..

[16] Park K.H., Kwak K.W., Nam S.H., Choi J.Y., Hyun S., Kim H.G., Kim S.H. (2015). Antibacterial activity of larval extract from the black soldier fly Hermetia illucens (Diptera: Stratiomyidae) against plant pathogens. J. Entomol. Zool. Stud..

[17] Teh C.H., Nazni W.A., Lee H.L., Fairuz A., Tan S.B., Sofian-Azirun M. (2013). In vitro antibacterial activity and physicochemical properties of a crude methanol extract of the larvae of the blow fly Lucilia cuprina. Med. Vet. Entomol..

[18] Li S., Ji H., Zhang B., Tian J., Zhou J., Yu H. (2016). Influence of black soldier fly (Hermetia illucens) larvae oil on growth performance, body composition, tissue fatty acid composition and lipid deposition in juvenile Jian carp (Cyprinus carpio var. Jian). Aquaculture.

[19] Bauer A.W., Kirby W.M.M., Sherris J.C., Turck M. (1966). Antibiotic Susceptibility Testing by a Standardized Single Disk Method. Am. J. Clin. Pathol..

[20] Thornsberry C., BALOWS A., Microbiology A.S., Hausler W.J., Herrmann K.L., Isenberg H.D., Shadomy H.J. (1991). Antimicrobial susceptibility testing: General considerations. Manual of Clinical Microbiology.

[21] Ordóñez R.M., Zampini I.C., Moreno M.I.N., Isla M.I. (2011). Potential application of Northern Argentine propolis to control some phytopathogenic bacteria. Microbiol. Res..

[22] Sledz W., Los E., Paczek A., Rischka J., Motyka A., Zoledowska S., Piosik J., Lojkowska E. (2015). Antibacterial activity of caffeine against plant pathogenic bacteria. Acta Biochim. Pol..

[23] Scrimgeour C., Shamsi I.H., Shamsi B.H., Jiang L. (2005). Chemistry of fatty acids. Bailey’s Industrial Oil and Fat Products.

[24] Ramos-Bueno R.P., González-Fernández M.J., Sánchez-Muros-Lozano M.J., García-Barroso F., Guil-Guerrero J.L. (2016). Fatty acid profiles and cholesterol content of seven insect species assessed by several extraction systems. Eur. Food Res. Technol..

[25] Caligiani A., Marseglia A., Sorci A., Bonzanini F., Lolli V., Maistrello L., Sforza S. (2019). Influence of the killing method of the black soldier fly on its lipid composition. Food Res. Int..

[26] Agoramoorthy G., Chandrasekaran M., Venkatesalu V., Hsu M.J. (2007). Antibacterial and antifungal activities of fatty acid methyl esters of the blind-your-eye mangrove from India. Braz. J. Microbiol..

[27] Sivakumar R., Jebanesan A., Govindarajan M., Rajasekar P. (2011). Larvicidal and repellent activity of tetradecanoic acid against Aedes aegypti (Linn.) and Culex quinquefasciatus (Say.) (Diptera:Culicidae). Asian Pac. J. Trop. Med..

[28] Choi W.H., Jiang M. (2014). Evaluation of antibacterial activity of hexanedioic acid isolated from Hermetia illucens larvae. J. Appl. Biomed..

[29] Kim S.A., Rhee M.S. (2016). Highly enhanced bactericidal effects of medium chain fatty acids (caprylic, capric, and lauric acid) combined with edible plant essential oils (carvacrol, eugenol, b-resorcylic acid, trans-cinnamaldehyde, thymol, and vanillin) against Escherichia coli O15. Food Control.

[30] Sugumaran M. (2002). Comparative Biochemistry of Eumelanogenesis and the Protective Roles of Phenoloxidase and Melanin in Insects. Pigment Cell Res..

[31] Park K.H., Zeon S.-R., Lee J.-G., Choi S.-H., Shin Y.K., Park K.-I. (2014). In vitro and in vivo efficacy of drugs against the protozoan parasite *Azumiobodo hoyamushi* that causes soft tunic syndrome in the edible ascidian *Halocynthia roretzi* (Drasche). J. Fish Dis..

[32] Soetemans L., Uyttebroek M., D’Hondt E., Bastiaens L. (2019). Use of organic acids to improve fractionation of the black soldier fly larvae juice into lipid- and protein-enriched fractions. Eur. Food Res. Technol..

[33] Choi W.H., Yun J.H., Chu J.P., Chu K.B. (2012). Antibacterial effect of extracts of hermetia illucens (diptera: Stratiomyidae) larvae against gram-negative bacteria. Entomol. Res..

[34] Ushakova N.A., Brodskii E.S., Kovalenko A.A., Bastrakov A.I., Kozlova A.A., Pavlov D.S. (2016). Characteristics of lipid fractions of larvae of the black soldier fly Hermetia illucens. Dokl. Biochem. Biophys..

[35] Ewald N., Vidakovic A., Langeland M., Kiessling A., Sampels S., Lalander C. (2020). Fatty acid composition of black soldier fly larvae (Hermetia illucens)—Possibilities and limitations for modification through diet. Waste Manag..

[36] Barroso F.G., Sánchez-Muros M.J., Segura M., Morote E., Torres A., Ramos R., Guil J.L. (2017). Insects as food: Enrichment of larvae of Hermetia illucens with omega 3 fatty acids by means of dietary modifications. J. Food Compos. Anal..

[37] Liland N.S., Biancarosa I., Araujo P., Biemans D., Bruckner C.G., Waagbø R., Torstensen B.E., Lock E.-J.J. (2017). Modulation of nutrient composition of black soldier fly (Hermetia illucens) larvae by feeding seaweed-enriched media. PLoS ONE.

[38] Meneguz M., Schiavone A., Gai F., Dama A., Lussiana C., Renna M., Gasco L. (2018). Effect of rearing substrate on growth performance, waste reduction efficiency and chemical composition of black soldier fly (Hermetia illucens) larvae. J. Sci. Food Agric..

[39] Spranghers T., Ottoboni M., Klootwijk C., Ovyn A., Deboosere S., De Meulenaer B., Michiels J., Eeckhout M., De Clercq P., De Smet S. (2017). Nutritional composition of black soldier fly (Hermetia illucens) prepupae reared on different organic waste substrates. J. Sci. Food Agric..

[40] Alanis A.J. (2005). Resistance to antibiotics: Are we in the post-antibiotic era?. Arch. Med. Res..

[41] Kotan R., Cakir A., Dadasoglu F., Aydin T., Cakmakci R., Ozer H., Kordali S., Mete E., Dikbas N. (2010). Antibacterial activities of essential oils and extracts of Turkish Achillea, Satureja and Thymus species against plant pathogenic bacteria. J. Sci. Food Agric..

[42] Shea K.M. (2003). Antibiotic resistance: What is the impact of agricultural uses of antibiotics on children’s health?. Pediatrics.

[43] Sumayo M.S., Kwon D.K., Ghim S.Y. (2014). Linoleic acid-induced expression of defense genes and enzymes in tobacco. J. Plant Physiol..

[44] Schlievert P.M., Peterson M.L. (2012). Glycerol Monolaurate Antibacterial Activity in Broth and Biofilm Cultures. PLoS ONE.

[45] Skřivanová E., Molatová Z., Marounek M. (2008). Effects of caprylic acid and triacylglycerols of both caprylic and capric acid in rabbits experimentally infected with enteropathogenic Escherichia coli O103. Vet. Microbiol..

[46] Cohen Y., Gisi U., Mosinger E. (1991). Systemic resistance of potato plants against Phytophthora infestans induced by unsaturated fatty acids. Physiol. Mol. Plant Pathol..

[47] Blechert S., Brodschelm W., Hölder S., Kammerer L., Kutchan T.M., Mueller M.J., Xia Z.Q., Zenk M.H. (1995). The octadecanoic pathway: Signal molecules for the regulation of secondary pathways. Proc. Natl. Acad. Sci. USA.

[48] Farmer E.E., Ryan C.A. (1992). Octadecanoid Precursors of Jasmonic Acid Activate the Synthesis of Wound-Inducible Proteinase Inhibitors. Plant Cell.

[49] Kumar P.P., Kumaravel S., Lalitha C. (2010). Screening of antioxidant activity, total phenolics and GC-MS study of Vitex negundo. Afr. J. Biochem. Res..

[50] Awa E.P., Ibrahim S., Ameh D.A. (2012). GC/MS Analysis and Antimicrobial activity of Diethyl ether fraction of Methanoolic extract from the Stem Bark of Annona senegalensis Pers. Int. J. Pharm. Sci. Res..

[51] Ouattara B., Simard R.E., Holley R.A., Piette G.J.P., Bégin A. (1997). Antibacterial activity of selected fatty acids and essential oils against six meat spoilage organisms. Int. J. Food Microbiol..

[52] Nair R.R. (2017). Agnihotra Yajna: A Prototype of South Asian Traditional Medical Knowledge. JAMS J. Acupunct. Meridian Stud..

[53] Rahuman A.A., Gopalakrishnan G., Ghouse B.S., Arumugam S., Himalayan B. (2000). Effect of Feronia limonia on mosquito larvae. Fitoterapia.

[54] Khalil A.S., Rahim A.A., Taha K.K., Abdallah K.B. (2013). Characterization of Methanolic Extracts of Agarwood Leaves. J. Appl. Ind. Sci..

[55] Duke J.A. (1992). Handbook of Biologically Active Phytochemicals and Their Activities.

[56] Chandrasekaran M., Kannathasan K., Venkatesalu V. (2008). Antimicrobial activity of fatty acid methyl esters of some members of chenopodiaceae. Z. Naturforsch. Sect. C J. Biosci..

[57] Enig M.G., Watson R.R. (1998). Lauric oils as antimicrobial agents: Theory of effect, scientific rationale, and dietary application as adjunct nutritional support for HIV-infected individuals. Nutrients and Foods in AIDS.

[58] Pinto M.E.A., Araújo S.G., Morais M.I., Sá N.P., Lima C.M., Rosa C.A., Siqueira E.P., Johann S., Lima L.A.R.S. (2017). Antifungal and antioxidant activity of fatty acid methyl esters from vegetable oils. An. Acad. Bras. Cienc..

[59] Sahin N., Kula I., Erdogan Y. (2006). Investigation of antimicrobial activities of nonanoic acid derivatives. Fresenius Environ. Bull..

[60] Chandrasekaran M., Senthilkumar A., Venkatesalu V. (2011). Antibacterial and antifungal efficacy of fatty acid methyl esters from the leaves of *Sesuvium portulacastrum* L.. Eur. Rev. Med. Pharmacol. Sci..

[61] Abou-Elela G.M., Abd-Elnaby H., Ibrahim H.A.H., Okbah M.A. (2009). Marine Natural Products and Their Potential Applications as Anti-Infective Agents. World Appl. Sci. J..

[62] McGaw L.J., Jäger A.K., Van Staden J. (2002). Antibacterial effects of fatty acids and related compounds from plants. S. Afr. J. Bot..

[63] Kabara J.J., Scherr G.H., Scherr G.H. (1986). Advances in Human Nutrition.

[64] Feldlaufer M.F., Knox D.A., Lusby W.R., Shimanuki H. (1993). Antimicrobial activity of fatty acids against Bacillus larvae, the causative agent of American foulbrood disease. Apidologie.

[65] Teh C.H., Nazni W.A., Nurulhusna A.H., Norazah A., Lee H.L. (2017). Determination of antibacterial activity and minimum inhibitory concentration of larval extract of fly via resazurin-based turbidometric assay. BMC Microbiol..

[66] Meziani S., Oomah B.D., Zaidi F., Simon-Levert A., Bertrand C., Zaidi-Yahiaoui R. (2015). Antibacterial activity of carob (*Ceratonia siliqua* L.) extracts against phytopathogenic bacteria Pectobacterium atrosepticum. Microb. Pathog..

[67] Canche-Escamilla G., Colli-Acevedo P., Borges-Argaez R., Quintana-Owen P., May-Crespo J.F., Cáceres-Farfan M., Yam Puc J.A., Sansores-Peraza P., Vera-Ku B.M. (2019). Extraction of phenolic components from an Aloe vera (Aloe barbadensis Miller) crop and their potential as antimicrobials and textile dyes. Sustain. Chem. Pharm..

[68] Soberón J.R., Sgariglia M.A., Dip Maderuelo M.R., Andina M.L., Sampietro D.A., Vattuone M.A. (2014). Antibacterial activities of Ligaria cuneifolia and Jodina rhombifolia leaf extracts against phytopathogenic and clinical bacteria. J. Biosci. Bioeng..

[69] Hong H., Lee J.H., Kim S.K. (2018). Phytochemicals and antioxidant capacity of some tropical edible plants. Asian Australas. J. Anim. Sci..

[70] Halldor T., Thormar H. (2011). Hilmarsson Hilmar Antimicrobial Lipids as Disinfectants, Antiseptics and Sanitizers. Lipids and Essential Oils as Antimicrobial Agents.

[71] Stulnig T.M., Huber J., Leitinger N., Imre E.M., Angelisová P., Nowotny P., Waldhäusl W. (2001). Polyunsaturated Eicosapentaenoic Acid Displaces Proteins from Membrane Rafts by Altering Raft Lipid Composition. J. Biol. Chem..

[72] Zheng C.J., Yoo J.S., Lee T.G., Cho H.Y., Kim Y.H., Kim W.G. (2005). Fatty acid synthesis is a target for antibacterial activity of unsaturated fatty acids. FEBS Lett..

